# Effect of Liuzijue Exercise Combined with Elastic Band Resistance Exercise on Patients with COPD: A Randomized Controlled Trial

**DOI:** 10.1155/2018/2361962

**Published:** 2018-06-11

**Authors:** Weibing Wu, Xiaodan Liu, Peijun Li, Ning Li, Zhenwei Wang

**Affiliations:** ^1^Department of Sports Medicine, Shanghai University of Sport, Shanghai, China; ^2^School of Rehabilitation Medicine, Shanghai University of Traditional Chinese Medicine, Shanghai, China; ^3^Department of Respiratory Medicine, Yue-Yang Hospital of Integrated Traditional Chinese and Western Medicine, Shanghai, China

## Abstract

**Objectives:**

This study aimed to investigate the effect of Liuzijue exercise combined with elastic band resistance exercise on patients with chronic obstructive pulmonary disease (COPD) to provide a convenient, safe, and cost-effective exercise.

**Methods:**

Subjects were randomly divided into the control group (CG), the Liuzijue exercise group (LG), and the Liuzijue exercise combined with elastic band resistance exercise group (LEG), with 20 patients in each group. The LG performed Liuzijue exercise six times a week (two exercise sessions in the hospital and four exercise sessions at home). The LEG includes Liuzijue exercise similar to the LG and elastic band resistance exercise three times a week, with elastic band exercise implemented after Liuzijue exercise. Spirometry, 6-minute walking test (6MWT), 30-second sit-to-stand test (30 s SST), handgrip strength test, and St. George's Respiratory Questionnaire (SGRQ) were performed at baseline and at the end of intervention.

**Results:**

After six-month intervention, the forced expiratory volume in 1 second (% predicted), 6-minute walking distance (6MWD), 6MWD%pred, 30 s SST, and SGRQ were significantly improved in the intervention groups (p < 0.01) and handgrip strength was increased significantly in the LG and LEG (p = 0.03 and p = 0.001, respectively). Furthermore, improvements in 6MWD and SGRQ were distinguished in the intervention groups compared with the CG (p < 0.01). No difference was significant in all of the outcomes between the LG and the LEG.

**Conclusions:**

The intervention program of Liuzijue exercise combined with elastic band resistance exercise and Liuzijue exercise only has beneficial effects on COPD patients especially in the aspect of exercise capacity and quality of life.

## 1. Introduction

Chronic obstructive pulmonary disease (COPD) is characterized by persistent and progressive airflow limitation, which impairs the exercise capacity and the quality of life in COPD patients [1]. In addition to respiratory symptom, skeletal muscle dysfunction as a prominent extra-pulmonary manifestation can appear in the early stage of COPD [2] and it is an independent risk factor for predicting the mortality of COPD patients [2, 3]. Moreover, skeletal muscle dysfunction can impair the exercise capacity and quality of life in patients with COPD [4] leading to a vicious cycle. Consequently, the treatment of skeletal muscle dysfunction as an important component in the rehabilitation of COPD has receiving more and more attentions.

Pulmonary rehabilitation is a comprehensive intervention, which includes but not limited to exercise training, education, self-management, and psychological support [5]. Of that resistance training (RT) can effectively increase muscle mass and enhance muscle strength and even reduce all-cause mortality in COPD patients [6]. Aerobic training (AT) has better effects on improving peak oxygen uptake (VO_2_ peak) and peak working capacity (Wpeak) and inducing skeletal muscle oxidation phenotype in COPD patients compared with RT [7, 8]. Moreover, combined exercise training could achieve a better effect than single training modalities in COPD patients [7, 9]. Thereby, a program including RT and AT is needed for the rehabilitation of COPD patients. Conventional exercise training usually conducted by various facilities, including treadmill, cycle ergometer, weight training machines, and other types of equipment [10–12], brings a huge burden to patients, patients' family, and social healthcare resources [13, 14]. Hence, convenient and cost-effective exercise training methods should be explored for COPD patients.

Elastic bands as alternative equipment to resistance training are economical, safe, convenient to carry, and free from site restrictions. Furthermore, previous studies have confirmed that elastic band resistance exercise can achieve similar effects in improving muscle strength and quality of life compared with RT using conventional equipment, and it has a better effect on improving functional exercise capacity [15]. Traditional Chinese health exercise as a low-intensity aerobic training intervention [16] is free from equipment and site restrictions and is widely loved by the elderly, of which Liuzijue is featured by diaphragmatic breathing and pursed lip breathing can make COPD patients breathe slowly and prevent the premature airway occlusion caused by rapid airflow to improve the abnormal breathing pattern of patients [16, 17]. In addition, Liuzijue is performed by expiration in producing six different sounds (“xu,” “he,” “hu,” “si,” “chui,” and “xi”) together with corresponding body movements, which not only is beneficial to the function of the auxiliary breathing muscles, but also can enhance the flexibility, coordination, and control capacity on neuromuscular of limbs to further improve exercise capacity in patients [18].

Previous studies mostly focus on the effects of single training form on COPD patients, while they rarely focused on the effects of Liuzijue exercise combined with elastic band resistance training. Among them, a multicenter, single-blind, randomized controlled trial found that performing Liuzijue for six-month significantly improved exercise endurance, quality of life, specific conductance, and dyspnea [16]. Moreover, Qigong training, AT, and respiratory training can effectively improve dyspnea in patients with moderate COPD, while Qigong training and AT are more advantageous in reducing upper respiratory tract infections [19]. It has been suggested that traditional Chinese health exercise has significant effects on improving the exercise capacity, dyspnea, quality of life, and immune function in patients with COPD. The study applied elastic band RT on COPD patients and concluded that only functional exercise capacity and muscle function (isokinetic muscle strength) increased significantly, but exercise endurance and quality of life did not change [20]. It suggested that both Liuzijue and elastic band RT have positive effects on COPD patients, but the effects are slightly different.

The aim of this study was to investigate the effect of Liuzijue combined with elastic band RT to provide convenient and cost-effective exercise training for COPD patients. This study hypothesized that Liuzijue combined with elastic band RT could significantly improve lung function, exercise capacity, skeletal muscle strength, and quality of life in patients with COPD. Furthermore, combined exercise training could achieve better effects on improving exercise capacity and skeletal muscle strength compared with single exercise.

## 2. Methods

### 2.1. Design

A single-blind, randomized controlled trial was conducted with randomized grouping, concealed allocation, and outcome assessor blinding. After baseline measurements, the patients were randomly allocated into three groups (the control group (CG), the Liuzijue exercise group (LG), and the Liuzijue exercise combined with elastic band resistance exercise group (LEG)) using computer-generated random numbers table at 1:1:1 ratio. The random numbers table was produced by an independent person. Then the random number is sealed in opaque envelopes and allocated to patients by an independent person. It was difficult to blind the exercise instructor because of the exercise intervention in this study, while the outcome assessor was blinded. The study design was approved by the Ethics Committee of Yue-Yang Integrative Medicine Hospital affiliated with Shanghai University of Traditional Chinese Medicine (Shanghai, China), and all of the patients signed the informed consent before the intervention.

### 2.2. Sample Size

The simple size calculation of this study was based on the 6MWD, which is commonly used to reflect the effects of pulmonary rehabilitation. According to the data used in the study by Ng et al. [21], an average change of 54 m in 6MWD between control group and intervention group was identified as the minimal significant difference, and the standard deviation (SD) of the measure was 57 m. To achieve a power of 0.8 and a significance level of 0.05 for difference detection, we conducted a sample size calculation in PASS 11.0 and the results showed that a minimum sample size for each group was 18 participants. To compensate for a possibility dropout rate of 20%, 65 patients should be included.

### 2.3. Patients

COPD patients were recruited from Yue-Yang Integrative Medicine Hospital affiliated with Shanghai University of Traditional Chinese Medicine from March 2014 to March 2015. Patients were included if they satisfied the following criteria: (1) they were diagnosed with COPD based on the 2013 Global Initiative for Chronic Obstructive Lung Disease (GOLD) and in grades II and III (grade II: 50%≤FEV1<80% pred; grade III: 30%≤FEV1<50% pred) [22]; (2) they were stable for 4 weeks without acute exacerbation before randomization; (3) they were between 40 and 80 years of age; (4) they had not engaged in any regular exercise (at least twice a week) within 6 months prior to the intervention. Patients with severe cardiovascular, hepatorenal, and hematopoietic system disease, psychosis, or patients participated in other clinical trials were excluded.

### 2.4. Intervention

All patients continued their prescribed medication treatment. Before the exercise intervention, patients were required to complete a symptom-limited exercise incremental test to determine the maximal exercise capacity of patients. The test was conducted on a motor-driven treadmill (ERS.2, Ergoline, German) with a comparative increases in workload through a constant increase in speed (0.2 mph) and an increase in inclination (0.5%-1%) each minute, similar to Holm et al. (2014) [23]. Moreover, the duration of the test was controlled between 8 and 12 min to avoid the impacts of subjective fatigue [24]. The test required the patient to achieve personal maximal tolerance exercise load or exercise termination indications. Reasons for the termination of the test included extreme fatigue; the patient's rhythm of movement which could not keep up with the target speed required by the test; obvious dyspnea, chest tightness, dizziness, and other symptoms; blood oxygen saturation decreased to 85% and arrhythmia; abnormal blood pressure and heart rhythm; blood pressure greater than 200/100 mmHg; or an electrocardiograph showing myocardial ischemia and arrhythmia [24, 25]. During the test, the heart rate of patients was monitored by a heart rate watch (Polar team^2^, Polar, Finland), and patients rate their dyspnea and fatigue according to Borg scale (CR10, category ratio scale) [26].

In Liuzijue exercise, the intensity target was to achieve a mean heart rate on 60–80% of the maximum heart rate and the patients were encouraged to reach Borg score 3-4. In elastic band exercise, the intensity target was to achieve a 50-60% of 1 repetition maximum (or 10–15 RM) of the target muscle group and it was monitored via heart rate and Borg score. This study used progressive Thera-band elastic resistance bands (Thera-Bands®, Hygenic Corporation, USA) for exercise. Elastic band resistance was distinguished according to the color in this trial: tan < yellow < red < green < blue < black < silver < gold, and the adjacent color resistance had a difference of between 20 and 30% [27].

#### 2.4.1. Control Group

Patients in control group only received pharmacologic treatment, and no exercise program has been performed.

#### 2.4.2. Liuzijue Exercise Group

Guidance was provided for the patients three times a week for two weeks before the intervention. During this period, patients were educated about the complete motions and essentials tips of Liuzijue, and the teaching videos and exercise logs were allocated to patients.

The program of LG consisted of three parts as follows: (1) 10 min of extension exercise focusing on limb joint motions; (2) 40 min of Liuzijue exercise according to the Health Qigong Liuzijue video compiled by Chinese Health Qigong Association [28] (it consists of six distinct exhaling sounds (“xu,” “he,” “hu,” “si,” “chui,” and “xi” and corresponding exercises of the upper and the lower limbs); one session of Liuzijue exercise was conducted for 12–15 min; and (3) 10 min of relaxation exercise mainly to adjust breathing and flapping and pulling muscles.

Exercise was performed two times in the hospital and four times at home per week for six months. Each exercise program required recording the entire process, including exercise duration, exercise time, location, intensity, etc.

#### 2.4.3. Liuzijue Exercise Combined with Elastic Band Resistance Exercise Group

Guidance was provided for the patients three times a week for two weeks before the intervention. Patients were educated about the practices of Liuzijue and the movement of elastic band resistance exercise, the teaching videos, and exercise logs were allocated to patients.

The program of LEG was presented as follows: (1) 10 min of extension exercise that focused on limb joint motions; (2) 20 min of Liuzijue exercise that was the same as the LG; (3) 20 min of elastic band resistance exercise based on elastic band gymnastics developed by Xiangya Hospital affiliated with Central South University, which consisted of four motions for the upper and lower limbs, respectively; all of the exercises were performed in 2 sets of 8–12 repetitions and the patients were allowed a rest period of 1–3 min between sets; the resistance exercises target the following muscles: shoulder and elbow flexion and extension muscles (deltoid, supraspinatus, latissimus dorsi, triceps brachii, and biceps brachii) and hip and knee flexion and extension muscles (gluteus maximus, gluteus medius, quadriceps femoris, hamstring); (4) 10 min of relaxation exercise mainly to adjust the patient's breathing. Patients were asked to exhale during the forced phase and inhale during the relaxed phase to avoid holding their breath during exercise.

The frequency of Liuzijue exercise was identical to that of the LG, and the frequency of elastic band exercise was performed twice in the hospital and once at home per week for six months. After exercising, the patients were required to record the whole exercise process.

### 2.5. Outcome Measures

Outcome measures were conducted within a week at baseline and immediately after intervention.

#### 2.5.1. Pulmonary Function Assessments

Pulmonary function assessments were performed according to the guidelines of the American Thoracic Society/European Respiratory Society (ATS/ERS) [29]. Spirometry was conducted on a spirometer (MS-Diffusion, Jaeger, Germany) in the pulmonary function room in Yue-Yang Hospital. Each patient was tested 3 times and the best value was used to for the analysis. Predicted normal values were calculated according to the prediction formulas embedded in spirometry system (**Table 1**).

#### 2.5.2. 6-Min Walking Test (6MWT)

The 6-min walking distance (6MWD) was assessed according to the guidelines of the ATS [30]. A straight line (30 m in length) was selected in the corridor of the respiratory department and a chair was placed at each end to signify the beginning and end of the testing area. Patients walked back and forth as quickly as possible. The predicted values were calculated according to the equations provided by Enright and Sherrill [31].

#### 2.5.3. 30 s Sit-to-Stand Test

The 30 s sit-to-stand test (30 s SST) was conducted as the reference testing method [32]. Patients sat in a chair with the backrest placed against the wall, and they crossed their hands on their opposite shoulders. Participants made their feet open as wide as their shoulders, with knee flexion at approximately 90°. Then participants were instructed to stand with a straight knee. The best numbers from the two 30 s SST completed by the subjects were recorded.

#### 2.5.4. Handgrip Strength Test

Handgrip strength was assessed using an electron dynamometer (WCS-10000 Electron Dynamometer, SHWQ Electron Corporation, China). We adjusted the dynamometer based on the size of the palm of the participants. During the test, the participants kept their trunk upright and shoulder joint at 30° of abduction with their elbow extended. They grasped the hand dynamometer to the maximal extent and finished the test when the value on the display screen was not increased. The patients were told before the test to avoid momentary impulses. Patients executed two repetitions with 30 s intervals of rest and the maximum reading value was recorded for data analysis.

#### 2.5.5. Quality of Life

Quality of life was evaluated with the St. George's Respiratory Questionnaire (SGRQ), consisting of symptom, activity, and impact domains and total [33]. The symptom, activity, impact, and total score were obtained by SGRQ software (Peking Union Medical College Respiratory Medicine Development, Chinese version). Each domain of the SGRQ with the lower values indicated a better quality of life.

### 2.6. Statistical Analysis

Statistical analysis was performed using the software package SPSS 20.0 (SPSS, Inc., USA). The continuous data were evaluated using normality and variance homogeneity tests. Continuous data were presented as mean ± standard deviation (M ± SD) and categorical data were described as frequencies (%). For continuous data, the within-group differences were analyzed using the paired sample* t* test and intergroup differences were analyzed using one-way analysis of variance (ANOVA) and post hoc multiple comparisons were performed using Bonferroni test. For categorical data, the difference was analyzed by the chi-square test. The statistically significant level was set at P ≤ 0.05 (2-tailed).

## 3. Results

**Figure 1:**
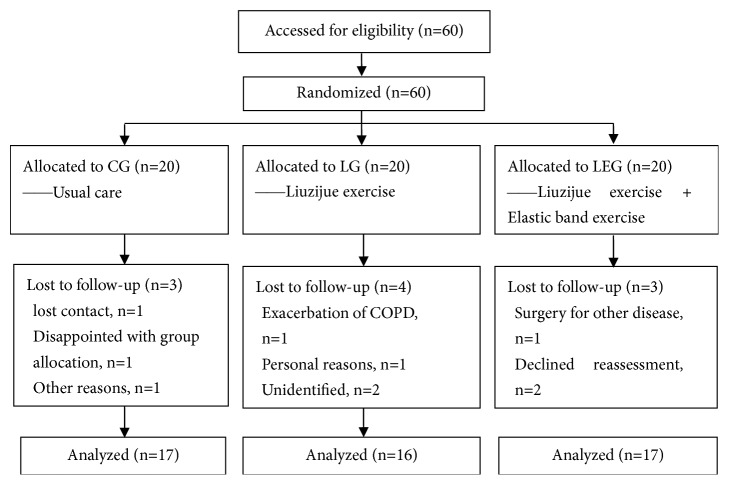
Study flow diagram. CG, control group; LG, Liuzijue exercise group; LEG, Liuzijue exercise combined with elastic band resistance exercise group.

Sixty patients with COPD were included in the study and randomized to CG, LG, and LEG. All patients received prescribed medication which was salmeterol/fluticasone inhalation (50 *μ*g/250 *μ*g, one inhalation per time, twice daily). During the study, 10 patients dropped out (3 in the CG, 4 in the LG, and 3 in the LEG) (**Figure 1**). Baseline characteristics for all three groups are shown in Table 2. No significant differences were detected among the groups at baseline, and the three groups have good comparability.

**Figure 2:**
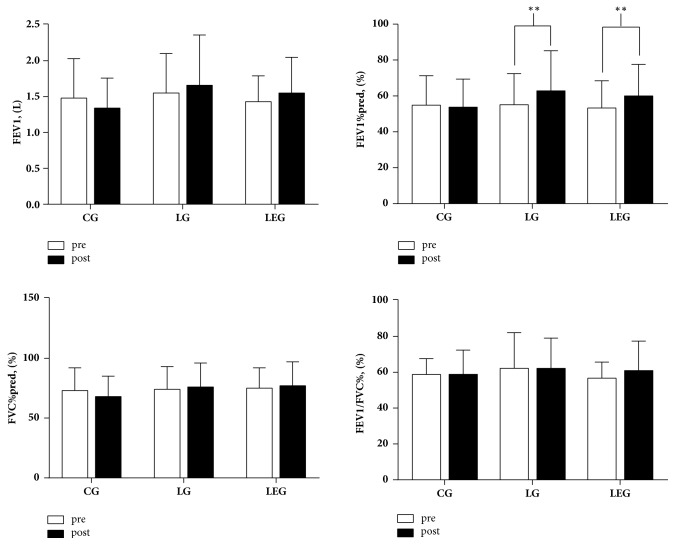
Pulmonary function in the CG, LG, and LEG groups after a six-month training period. CG, control group; LG, Liuzijue exercise group; LEG, Liuzijue exercise combined with elastic band resistance exercise group; FEV1, forced expiratory volume in 1 second; FVC, forced volume capacity.** Notes: **^*∗∗*^p < 0.05 within-group comparisons.

After six months of intervention, the FEV1%pred improved significantly (p < 0.01) in the LG and LEG patients (**Figure 2**). While the changes of FEV1 in LG and LEG have no statistical significance, the mean improvements of FEV1 in LG and LEG (0.11L and 0.12L, respectively) have been achieved the minimal clinically important difference (MCID) of 0.1 L [34]. There were no significant differences concerning MMEF25-75%, FEV1%pred, FVC%pred, and FEV1/FVC% among the three groups (**Table 3**).

**Figure 3:**
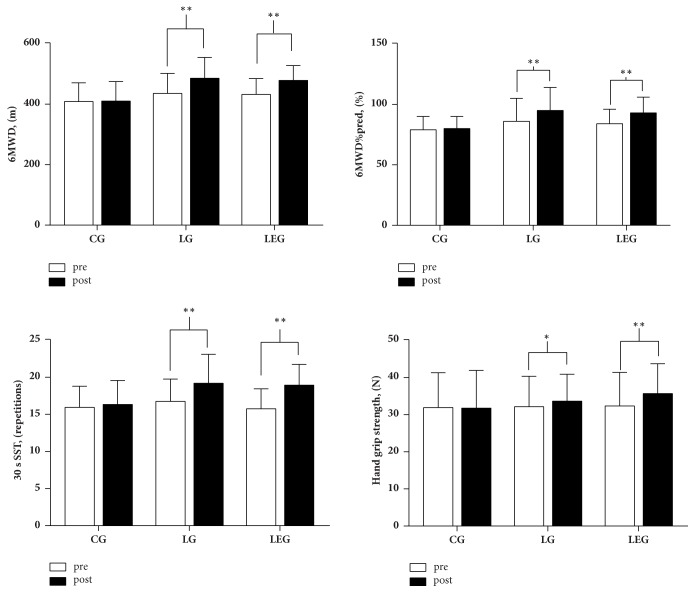
The 6MWD, 6MWD%pred, 30 s SST, and hand grip strength in the CG, LG, and LEG groups after a six-month training period. CG, control group; LG, Liuzijue exercise group; LEG, Liuzijue exercise combined with elastic band resistance exercise group; 6MWD, 6-min walking distance; 6MWD%pred, 6-min walking distance as a percentage of the predicted value; 30 s SST, 30 s sit-to-stand test.** Notes: **^*∗*^p < 0.05 within-group comparisons and ^*∗∗*^p< 0.01 within-group comparisons.

The mean improvements of 6MWD in the LG and LEG (LG: 50.12 m, LEG: 46.85 m) exceed a MCID of 30 m [35]. While the 6MWD and 6MWD%pred improved significantly in the LG and LEG after the intervention, no significant difference was found between the two groups (**Table 3**). In addition, the improvement in the LG and the LEG was significantly greater than in the CG (6MWD: p = 0.003 and p = 0.006, respectively; 6MWD%pred: p = 0.01 and p = 0.029, respectively). In addition, 30 s SST increased in the LG and the LEG (p = 0.001 and p < 0.001, respectively) (**Figure 3**). The mean improvements of 30 s SST in the LG and LEG (LG: 2.44 repetitions, LEG: 3.18 repetitions) may achieve the level of MCID, while the MCID of 1-min SST was 3 times [36]. There were significant differences in the 30 s SST among the three groups (p = 0.031), whereas there were no significant differences in every two-group comparisons. Similarly, handgrip strength increased significantly in the LG and the LEG (p = 0.03 and p = 0.001, respectively) (**Figure 3**). The mean improvements of handgrip strength in LG and LEG were 4% and 10%. While a minimum clinical improvement in muscle strength was set at 15% according to a previous study [14].

**Figure 4:**
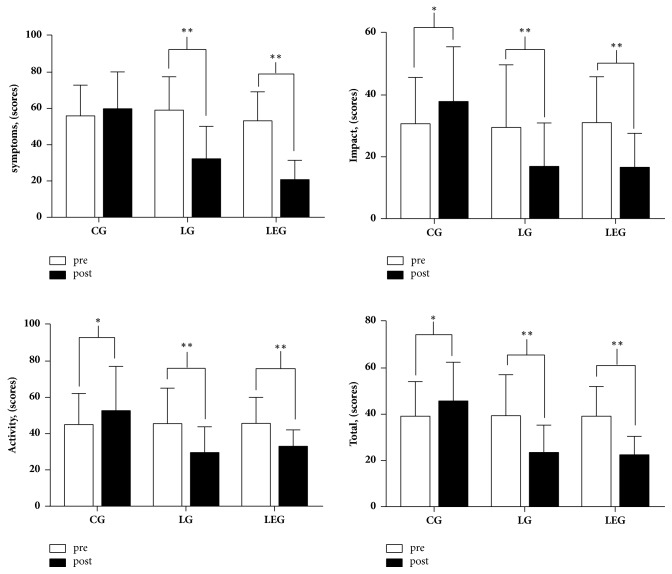
Domains of the St. George's Respiratory Questionnaire in the CG, LG, and LEG groups after the six-month training period. CG, control group; LG, Liuzijue exercise group; LEG, Liuzijue exercise combined with elastic band resistance exercise group.** Notes: **^*∗*^p < 0.05 within-group comparisons, and ^*∗∗*^p< 0.01 within-group comparisons.

The total score and three domains scores of SGRQ in the CG were increased, while the impact and activity score increased significantly (p < 0.05) and the SGRQ total score and subdomain score were decreased (p < 0.01) significantly in the LG and LEG (**Figure 4**). The mean improvements of each domain of SGRQ in the LG and LEG exceed the MCID of 4 points [33]. Intergroup comparisons showed that the improvement of the SGRQ score in the LG and LEG was significantly different when compared with the CG (p < 0.01), whereas the LG and LEG had no differences (**Table 4**).

## 4. Discussion

The present study showed that the program of Liuzijue exercise combined with elastic band resistance exercise results in improvements in pulmonary function, exercise capacity, and quality of life in patients with II to III grade COPD and has advantages in improving 6MWD and 30 s SST among the three groups. The deficient of statistical significance between LE and LEG may be attributed to the outcome measurements major reflect aerobic capacity and the low proportion of elastic band resistance exercise of the combined program induces insufficient effects on muscle.

In the aspect of the completion rate, there was a slight difference compared with previous studies, while a study applied with the Liuzijue exercise program was 94% [16] and a study applied with the RT program was 76% [15]. The differences may be related to the exercise type, time, intensity, whether the exercise was supervised, and the degree of supervision. Similar to the design of present study, an intervention preformed elastic band resistance exercise three times a week (once in the hospital, twice at home alone) in COPD patients, with only 56% of the patients completing it for 12 weeks [37]. This could be attributed to the intolerance of long-term resistance exercise alone or the lack of perception of the effect with patients, as well as the low extent of supervised exercise. The high completion rate of the present study further confirmed that Liuzijue exercise and elastic band resistance exercise are simple and easy to learn exercise.

Pulmonary function, as an important objective index to reflecting the severity of COPD, was progressively declining and difficult to delay with medication alone [38]. Pulmonary rehabilitation can effectively delay the decline of pulmonary function and improve exercise capacity in COPD patients [39]. The mentioned above can be found in present study also, that patients in CG showed a decrease in FEV1, while in LG and LEG they showed an increase of >0.1 L in FEV1 and a significant increase in FEV1%pred. Previous studies demonstrated that pulmonary function improved after traditional Chinese health exercise, whereas pursed lip breathing, abdominal breathing, and walking did not improve significantly [38]. A recent meta-analysis further confirmed the effects of traditional Chinese health exercise in COPD patients [40]. This may be explained by the fact that traditional Chinese exercise not only includes respiratory exercise, but also operates in tandem with upper and lower limb exercise to further strengthen the role of auxiliary respiratory muscles. In present study, COPD patients in LG and LEG groups have significantly increased FEV1%pred and the improvement of FEV1 achieved a minimal clinical significance difference. It demonstrated that both protocols can effectively improve pulmonary function, and elastic band resistance exercise did not contribute to additional effects on patients. Similar to the results, previous studies that adopted resistance exercise and combined exercise have demonstrated a significant improvement of muscle strength, while VO_2_ peak and peak working capacity improved in aerobic exercise and combined exercise groups [7].

In present study, patients with grade II-III chronic obstructive pulmonary disease presented 6MWD at baseline that an average of 83% of the predicted distances was similar to previous study [15]. After training, the two intervention programs reached an average of 94% of the predicted 6MWD, which indicates the training effects of both programs in improving functional exercise capacity. A previous study revealed that the degree of improvement in walking capacity in COPD patients is influenced by age and baseline conditions; that is, patients over 65 years of age who have 6MWD baseline values above 350 m have less potential to increase [41]. Another study found an increased walking distance of 20.5 m (321.5 ± 15.5 m, p = 0.02), when applying the Liuzijue exercise on COPD patients aged about 70 years, and the baseline value of 6MWD was 301 ± 10.9m [16]. However, the present study observed that the significant improvement of walking capacity (485.75 ± 68.91 m, p = 0.001) was shown in the Liuzijue exercise group in which the baseline value of 6MWD was 435.63 ± 66.27 m in COPD patients who were about 67 years of age. It suggested that the walking capacity of COPD patients may not only be affected by age and baseline values, but also be affected by pulmonary function, aerobic capacity, and other factors. In addition, the improvement of 6MWD in COPD patients also exceeded the minimum clinical importance difference of 30 m [35]. Interestingly, a previous research showing no significant difference in increasing 6MWD between aerobic combined resistance exercise and aerobic exercise [9], while another study found that elastic band resistance exercise appears to improve 6MWD in COPD patients better than conventional resistance exercise (elastic band: 73 m and conventional: 42 m, respectively) [15]. However, it is difficult to determine if elastic band resistance exercise combined with aerobic exercise has additional effects on 6MWD compared with aerobic exercise due to the great heterogeneity of the two studies in terms of research type and study intervention. In addition, the different proportion of elastic band resistance exercise in training program may be the reason why there was no significant difference between the two groups.

30 s SST as a more suitable method for patients with severe COPD patients [32] was used to assess the exercise capacity, and the reliability and validity have been confirmed [42]. Studies have demonstrated that different exercise methods can effectively improve the capacity of SST completed by COPD patients [42, 43] but it is unclear which method is superior. Previous studies found that strength training-based pulmonary rehabilitation has advantages on improving 30 s SST in COPD patients [42], while in present study an increase was detected in both LG and LEG patients. According to the values of MCID in 1-min SST, the average improvement in LG and LEG (2.44 repetitions and 3.18) in present study may have clinical significance. In addition, the intervention effect of 30 s SST in COPD patients was significantly different among the three groups; however, there was no difference between any two of the groups. It is assumed that the overall variation was mainly due to intragroup differences rather than intergroup differences, and the results may attribute to the smaller size of sample when compared any two of the groups.

In addition, upper limb activities are an important part of daily life. Upper limb activities in daily life may affect the normal breathing of COPD patients, exacerbating pulmonary hyperinflation [44]. Moreover, upper extremity muscle strength is associated with exercise capacity and quality of life in COPD patients [45], and elbow activity is associated with pulmonary function and respiratory muscle strength in COPD patients [46]. Handgrip strength as a good indicator of muscular strength in the upper arms is closely related to the prognosis of COPD patients [47]. The aforementioned studies suggested that upper extremity muscle strength and function play an important role in the progression of COPD patients. In present study, LG and LEG patients have similarly increased in the handgrip strength, which is not consistent with previous findings in which researchers determined that resistance exercise and combined exercise have the advantage of enhancing skeletal muscle strength [8]. In addition, the improvements in LG and LEG (4% and 10%, respectively) did not achieve the MCID of 15% raised by Chen et al [14]. It may be attributed to the different methods used to evaluate muscle strength, different muscles assessed, and different training program. That isometric and isokinetric measurement systems were used to assess muscle function of leg by Chen et al.; electron dynamometer was used to assess handgrip strength in present study. Although no significant difference was detected between LG and LEG, the improvement in LEG has a larger extent compared with LG. It suggested that resistance training has advantages on improving muscle strength.

Previous studies have shown that different exercise programs can significantly improve the quality of life in patients with COPD, and combined exercise has a better effect [7]. In this study, although LEG program seemed to additionally decrease the symptom and impact scores of SGRQ, there was no difference between the intervention groups. Consistently, SGRQ scores were decreased after interventions and exceeded the minimum clinically important difference of 4 scores [33].

The main advantage of this study was that it combined traditional Chinese exercise with elastic band resistance exercise; both are safe, convenient, and effective exercise program for the rehabilitation of COPD patients. In addition, interventions in this study mainly focused on family exercise interventions, which can reduce the burden of medical resources. However, there are some limitations of this study. First, it is difficult to demonstrate the role of elastic band resistance exercise in a combined exercise program, as this study only compared the effects of combined exercise with Liuzijue exercise. Therefore, future studies are needed to improve the research design to clarify the role of elastic band resistance exercise in a combined exercise program for COPD patients. Second, the effect of elastic band resistance exercise might be underestimated, due to the smaller proportion of elastic band resistance exercise being less than Liuzijue exercise. Third, the sample size was relatively small, no quantitative data (such as isokinetic dynamometry) to reflect the limbs skeletal muscle function. Lastly, the effect of upper limb resistance exercise is difficult to confirm due to the lack of assessment indicators of the upper limb muscle group function and strength. Therefore, future studies should conduct with large sample and include relevant quantitative indicators that reflect the skeletal muscle function and strength of upper and lower extremities in order to comprehensively investigate the effect of Liuzijue exercise combined with elastic band resistance exercise.

## 5. Conclusion

Liuzijue exercise combined with elastic band resistance exercise can significantly improve the pulmonary function, exercise capacity, muscle strength, and quality of life in COPD patients and provide a convenient and effective combined exercise program for COPD patients. However, Liuzijue exercise combined with elastic band resistance exercise had no additional effects compared to Liuzijue exercise alone, which could be attributed to the small proportion of elastic band resistance exercise in this study. Therefore, future studies should further explore whether combined exercise can achieve additional effects compared to a single type of exercise and its optimal proportion.

## Figures and Tables

**Table 1:** Predictive equation for FEV1 and FVC.

**Indicators**	**Gender**	**Formula**
FEV1	Male	0.043*∗*(height*∗*100)-0.029*∗*age-2.49
	Female	0.0395*∗*(height*∗*100)-0.025*∗*age-2.6
FVC	Male	0.0576*∗*(height*∗*100)-0.026*∗*age-4.34
	Female	0.0443*∗*(height*∗*100)-0.026*∗*age-2.89

FEV1, forced expiratory volume in 1 second; FVC, forced volume capacity. **Notes:** height was expressed as meter.

**Table 2:** Baseline characteristics.

**Characteristics**	**CG (n = 17)**	**LG (n = 16)**	**LEG (n = 17)**	**P-value** ^**∗**^
**Age (years)**	66 ± 9	67 ± 8	64 ± 8	0.71
**Sex (male/female, n)**	14/3	14/2	13/4	0.71
**Years of COPD**	12 ± 4	13 ± 4	12 ± 4	0.83
**BMI (kg/m** ^**2**^ **)**	22.65 ± 3.76	22.5 ± 3.41	23.12 ± 3.25	0.86
**Smoking history (yes/no, n)**	14/3	14/2	13/4	0.71
**Smoking status, n **(%)**:**				
** Current smoker**	3/17.6%	1/6.2%	2/11.8%	0.76
** Ex-smoker**	11/64.7%	13/81.2%	11/64.7%	
** Never smoke**	3/17.6%	2/12.5%	4/23.5%	
**Stage of COPD, n **(%)				
** Grade II**	13/76.5%	11/68.8%	13/76.5%	0.85
** Grade III**	4/23.55	5/31.2%	4/23.5%	

CG, control group; LG, Liuzijue exercise group; LEG, Liuzijue exercise combined with elastic-band resistance exercise group; COPD, chronic obstructive pulmonary disease; BMI, body mass index.

**Notes:** data was expressed as mean ± SD or n (%). The one-way analysis of variance (ANOVA) was used to compare the age, years of COPD, and BMI among groups, and the chi-square test was used to compare the sex, smoking history, smoking status, and COPD stage among groups. The significance level was set at P<0.05. ^*∗*^P-value for the comparison of outcome variables among groups.

**Table 3:** Comparisons of pulmonary function and exercise capacity.

	**CG (n = 17)**		**LG (n = 16)**		**LEG (n = 17)**		**P** ^**a**^	**P** ^**b**^	**P** ^**c**^
	**Pre**	**Post**	**Pre**	**Post**	**Pre**	**Post**			
**FEV1, L**	1.48 ± 0.55	1.34 ± 0.42	1.55 ± 0.55	1.66 ± 0.7	1.43 ± 0.36	1.55 ± 0.5	0.82	1	1
**MMEF25-75**%**, L/s**	0.99 ± 0.54	0.97 ± 0.54	1 ± 0.64	1.03 ± 0.64	0.94 ± 0.63	1 ± 0.68	0.962	1	1
**FEV1**%**pred, **%	55 ± 16	54 ± 16	55 ± 17	63 ± 22^*∗∗*^	53 ± 15	60 ± 18^*∗∗*^	0.36	0.49	1
**FVC%pred, **%	73 ± 19	68 ± 17	74 ± 19	76 ± 20	75 ±17	77 ± 20	0.33	0.64	0.56
**FEV1/FVC**%, %	59 ± 9	59 ± 14	62 ± 20	62 ± 17	57 ± 9	61 ± 16	0.82	1	1
**6MWD, m**	408.71 ± 61.88	410.41 ± 64.35	435.63 ± 66.27	485.75 ± 68.91^*∗∗*^	432.06 ± 52.78	478.91 ± 48.93^*∗∗*^	0.01	0.003	0.006
**6MWD**%**pred, **%	79 ± 11	80 ± 10	86 ± 19	95 ± 19^*∗∗*^	84 ± 12	93 ± 13^*∗∗*^	0.006	0.01	0.029
**30s SST, repetitions**	15.94 ± 2.86	16.35 ± 3.22	16.75 ± 3.02	19.19 ± 3.9^*∗∗*^	15.76 ± 2.68	18.94 ± 2.79^*∗∗*^	0.031	0.054	0.08
**Handgrip strength, N**	31.92 ± 9.38	31.75 ± 10.18	32.14 ± 8.20	33.64 ± 7.26^*∗*^	32.35 ± 9.04	35.69 ± 8.05^*∗∗*^	0.42	1	0.57

CG, control group; LG, Liuzijue exercise group; LEG, Liuzijue exercise combined with elastic-band resistance exercise group; FEV 1, forced expiratory volume in 1 second; FVC, forced volume capacity; MMEF, maximal midexpiratory flow; 6MWD, 6-min walking distend; 6MWD%pred, 6-min walking distance as a percentage of the predicted value; 30s SST, 30s sit-to-stand test.

**Notes:** data was expressed as mean ± SD. The paired sample t-test was used for comparisons within groups, the one-way analysis of variance (ANOVA) was used for comparisons among groups, and post hoc multiple comparisons were performed using Bonferroni test. The significance level was set at P<0.05. None of significant difference has been found between the two training groups.

^*∗*^p < 0.05, comparisons are significant within groups. ^*∗∗*^p < 0.01, comparisons are significant within groups.

^a^P values for the comparison of outcome variables from baseline to 6-month among three groups.

^b^P values for the comparison of P values between CG and LG.

^c^P values for the comparison of P values between CG and LEG.

**Table 4:** Comparisons of quality of life assessed by SGRQ.

	**CG (n=17)**		**LG (n=16)**		**LEG (n=17)**	
	**Pre**	**Post**	**Pre**	**Post**	**Pre**	**Post**
**Symptom, score**	55.94 ± 16.92	59.88 ± 20.32	59.13 ± 18.45	32.31 ± 17.86^*∗∗*^	53.18 ± 15.99	20.82 ± 10.64^*∗∗*^
**Impact, score**	30.71 ± 15.03	37.94 ± 17.68^*∗*^	29.50 ± 20.29	16.94 ± 14.01^*∗∗*^	31.06 ± 14.86	16.65 ± 10.99^*∗∗*^
**Activity, score**	45 ± 17.22	52.71 ± 24.43^*∗*^	45.56 ± 19.54	29.63 ± 14.17^*∗∗*^	45.71 ± 14.33	33 ± 9.11^*∗∗*^
**Total, score**	39.18 ± 14.96	45.76 ± 16.77^*∗*^	39.38 ± 17.69	23.5 ± 11.82^*∗∗*^	39.18 ± 12.82	22.47 ± 7.97^*∗∗*^

SGRQ, St. George's Respiratory Questionnaire; CG, control group; LG, Liuzijue exercise group; LEG, Liuzijue exercise combined with elastic-band resistance exercise group.

**Notes:** data was expressed as mean ± SD. The paired samples t-test was used for comparisons within group, and the one-way analysis of variance (ANOVA) and Bonferroni test were used for comparisons among groups. The significance level was set at P<0.05. Difference was significant between the control group and the intervention group in all domains of SGRQ (P < 0.01), while no significant difference has been found between the two training groups.

^*∗*^p < 0.05, comparisons are significant within groups. ^*∗∗*^p < 0.01, comparisons are significant within groups.

## Data Availability

The original data used to support the findings of this study are available from the corresponding author upon request.
